# A Pilot Serum Metabolomics Reveals Mitochondrial Dysfunction and Identifies Methylguanidine as a Potential Diagnostic Biomarker for ATAAD with Mesenteric Malperfusion Syndrome

**DOI:** 10.3390/metabo16040240

**Published:** 2026-04-01

**Authors:** Junyi Wen, Weiliang Zheng, Lijun Sun, Lin Lu, Zhi Lin, Lulu Chen, Hua Peng, Yan Wang

**Affiliations:** 1 School of Medicine, Xiamen University, Xiamen 361005, China; w2199652053@163.com (J.W.); lulinusa@163.com (L.L.); proflinz@163.com (Z.L.); chenluluxmu@163.com (L.C.); 2 Division of Cardiovascular Surgery, Xiamen Cardiovascular Hospital, Xiamen University, Xiamen 361006, China; zwliang1980@163.com (W.Z.); sunlijun6172021@163.com (L.S.); 3 Division of Cardiovascular Medicine, School of Medicine, Xiamen Cardiovascular Hospital, Xiamen University, Xiamen 361006, China

**Keywords:** mesenteric malperfusion syndrome, metabolomics, lipidomics, mitochondria metabolism, methylguanidine, aortic dissection, biomarker

## Abstract

Background: Acute type A aortic dissection complicated by mesenteric malperfusion syndrome (ATAAD-MMPS) is a highly lethal emergency with diagnostic challenges due to rapid progression and non-specific symptoms. This pilot study aimed to characterize the serum metabolomic and lipidomic alterations specific to ATAAD-MMPS and identify potential early diagnostic biomarkers. Methods: Serum samples from healthy controls, patients with uncomplicated ATAAD, and patients with ATAAD-MMPS were analyzed using targeted metabolomics and lipidomics. Multivariate statistical analyses were performed to discriminate between groups and identify differentially abundant metabolites and lipids. Pathway analysis was conducted to explore underlying pathological mechanisms. Results: Metabolomic profiles clearly distinguished ATAAD-MMPS from uncomplicated ATAAD, whereas lipidomic changes were primarily associated with ATAAD itself rather than the presence of mesenteric malperfusion. Metabolic pathway analysis revealed significant perturbations in the citric acid cycle, suggesting mitochondrial involvement as a potential pathological feature. Notably, methylguanidine was uniquely and markedly elevated in the ATAAD-MMPS group, demonstrating potential diagnostic value in distinguishing this lethal complication from uncomplicated ATAAD in this exploratory cohort (AUC = 0.923). Conclusions: This pilot study identifies distinct metabolic signatures associated with mesenteric malperfusion in ATAAD, with mitochondrial metabolic perturbations emerging as a potential contributing mechanism. Methylguanidine represents a candidate early diagnostic biomarker for ATAAD-MMPS, warranting validation in larger prospective studies. These findings provide a foundation for improved diagnostic strategies for this devastating condition.

## 1. Introduction

Acute type A aortic dissection (ATAAD) is a catastrophic cardiovascular emergency associated with extremely high morbidity and mortality. The estimated annual incidence ranges from 5 to 30 cases per million population, with a male predominance, and mortality increases by 1–2% per hour after symptom onset [1]. Independent predictors of poor outcomes include advanced age, malperfusion, low left ventricular ejection fraction, prior cardiac surgery, preoperative mechanical ventilation or resuscitation, and concomitant coronary artery bypass grafting [2]. Malperfusion syndrome occurs in approximately 25% of ATAAD patients and significantly worsens prognosis. Among all malperfusion subtypes, mesenteric malperfusion is particularly lethal, with an adjusted odds ratio for in-hospital mortality of 4.84 (*p* < 0.001) [3] and reported mortality rates as high as 60–75% [4]. Despite current guidelines recommending early surgical intervention as a Class IA strategy for ATAAD complicated by mesenteric malperfusion syndrome (MMPS) [1], the underlying pathophysiological mechanisms remain poorly understood, and effective early diagnostic biomarkers are lacking [5].

Mesenteric malperfusion initiates a cascade of intestinal pathologies, including reduced mucosal perfusion, intramucosal acidosis, mucosal edema (manifesting as bowel wall thickening), ischemia, epithelial dysfunction, and increased intestinal permeability [6]. This disruption of the intestinal barrier facilitates bacterial translocation—the passage of bacteria and their products, such as lipopolysaccharide (LPS), into the circulation. Elevated circulating levels of these microbial components activate monocytes and macrophages, triggering the release of pro-inflammatory mediators and contributing to systemic inflammation. This mechanism has been implicated in aortic dissection [7] and other cardiovascular conditions. For instance, gut microbiota translocation and associated alterations in microbial metabolites have been shown to exacerbate acute myocardial ischemia by modulating intestinal permeability, oxidative stress, and energy metabolism [8].

Multi-omics approaches, including metabolomics and lipidomics, have revolutionized the study of human diseases by enabling comprehensive characterization of molecular phenotypes [9]. Metabolomics, the high-throughput analysis of endogenous metabolites, offers a powerful tool for capturing phenotypic changes and identifying novel biomarkers for early disease prediction, diagnosis, and subtyping [10]. Lipidomics, a specialized branch of metabolomics, provides detailed insights into lipid metabolism alterations that often reflect membrane remodeling, energy homeostasis, and inflammatory states [11]. The integration of these complementary omics layers has proven particularly valuable in cardiovascular research, where metabolic dysregulation plays a central role in disease pathogenesis [12,13]. For example, Wang et al. demonstrated that the gut microbiota-derived metabolite hippuric acid plays a key role in intestinal ischemia–reperfusion-induced acute lung injury [14]. Circulating levels of microbial metabolites such as trimethylamine N-oxide, choline, and crotonylbetaine have been independently associated with heart failure risk [15].

In the context of aortic dissection, emerging evidence suggests significant metabolic perturbations. Zeng et al. identified via untargeted metabolomics that certain metabolites correlate with disease severity [16]. Furthermore, lysophosphatidylcholines (LPCs) and sphingolipids—including sphingosine, phytosphingosine, and ceramide—are significantly altered in ATAAD patients, and their combined profile may serve as a potential biomarker for diagnosing and distinguishing Stanford type A from type B dissections [17]. These metabolic perturbations likely reflect the distinct clinical and histopathological features observed during dissection progression [18]. Despite these growing insights into the metabolic basis of aortic dissection, research specifically addressing the metabolic alterations associated with malperfusion, particularly mesenteric malperfusion, remains surprisingly limited. To date, no studies have reported early diagnostic biomarkers for ATAAD complicated by mesenteric malperfusion, and the pathophysiological mechanisms linking intestinal ischemia to systemic metabolic changes have yet to be elucidated.

Therefore, this exploratory pilot study aimed to: (1) characterize the serum metabolomic and lipidomic signatures specific to ATAAD with mesenteric malperfusion; (2) identify candidate early diagnostic biomarkers for ATAAD-MMPS; and (3) explore underlying pathological mechanisms through pathway analysis. We hypothesized that ATAAD-MMPS would exhibit distinct metabolic alterations compared to uncomplicated ATAAD, with some of these metabolites serving as potential biomarkers for early detection of this lethal complication. The findings from this pilot investigation are intended to generate hypotheses and provide a foundation for future validation studies.

## 2. Materials and Methods

### 2.1. Human Specimens and Selection Criteria

This study was conducted in accordance with the ethical principles of the Declaration of Helsinki and was approved by the Medical Ethics Committee of the Cardiovascular Hospital affiliated with Xiamen University (Approval No. K02202406). From a retrospective review of 568 patients with acute type A aortic dissection (ATAAD) admitted to the Xiamen Cardiovascular Hospital, Xiamen University, between January 2019 and January 2024, 28 patients were identified with clinically significant mesenteric malperfusion syndrome (MMPS). These patients presented with typical symptoms of mesenteric ischemia, including nausea, vomiting, abdominal pain, and abdominal distension, along with corresponding imaging findings on computed tomography angiography (CTA) [1]. Patients with ATAAD-MMPS were enrolled in this study according to the following exclusion criteria: (1) hereditary aortic diseases, autoimmune disorders, or connective tissue diseases; (2) familial or recurrent aortic coarctation; (3) prior history of cardiac or major vascular surgery; (4) concomitant comorbidities including acute myocardial infarction, acute cerebral infarction, severe hepatic or renal dysfunction, and malignant tumors; and (5) pre-existing gastrointestinal conditions (e.g., tumors, irritable bowel syndrome, or intra-abdominal pathologies). Patients with ATAAD-MMPS were matched with both uncomplicated ATAAD patients and healthy controls based on age, sex, and history of hypertension or diabetes mellitus. Clinical data collected upon admission included laboratory test results obtained within 24 h and imaging examinations (color Doppler ultrasound and computed tomography angiography [CTA]).

### 2.2. Serum Sample Collection and Preparation of Omics Samples

Whole blood samples were collected into serum separation tubes with gray caps (KDL, Shanghai, China) and immediately refrigerated at 4 °C to allow clotting. Following clot formation, samples were centrifuged at 3000 rpm for 15 min at room temperature. The resulting serum was carefully aliquoted and stored at −80 °C until further analysis. Targeted metabolomic and lipidomic profiling was performed by Applied Protein Technology (Shanghai, China). The complete lists of detected metabolites and lipids are provided in the Supplementary Materials.

For metabolomic analysis, metabolite extraction was achieved by adding 400 µL of ice-cold methanol/acetonitrile (1:1, *v*/*v*) to each serum sample to precipitate proteins, followed by thorough vortexing. Stable isotope-labeled internal standards were incorporated into the extraction solvent for absolute quantification. The mixture was transferred to a new microcentrifuge tube and centrifuged at 14,000× *g* for 20 min at 4 °C. The supernatant was collected and dried under vacuum centrifugation. Prior to liquid chromatography–mass spectrometry (LC—MS) analysis, the dried extracts were reconstituted in 100 µL of acetonitrile/water (1:1, *v*/*v*), vortexed, and centrifuged at 14,000× *g* for 15 min at 4 °C, after which the clarified supernatant was injected.

For lipidomic analysis, lipids were extracted using the methyl tert-butyl ether (MTBE) method. Briefly, serum samples were first mixed with 200 µL of methanol, followed by the addition of 10 µL of internal lipid standards and 800 µL of MTBE. The mixture was thoroughly vortexed, sonicated at 4 °C for 20 min, and then allowed to stand at room temperature for 30 min. Subsequently, 200 µL of MS-grade water was added to induce phase separation. After vortexing, the samples were centrifuged at 14,000× *g* rpm for 15 min at 4 °C. The upper organic phase containing lipids was carefully collected and evaporated to dryness under a gentle stream of nitrogen. For LC—MS analysis, the lipid extracts were reconstituted in 200 µL of isopropanol/acetonitrile (9:1, *v*/*v*), vortexed, and centrifuged at 14,000× *g* rpm for 15 min at 4 °C, after which the supernatant was injected.

### 2.3. LC-MS/MS Method and Data Processing

For metabolomic profiling, chromatographic separation was performed using an ultra-high performance liquid chromatography (UHPLC) system (1290 Infinity LC, Agilent Technologies, Santa Clara, CA, USA) coupled to a QTRAP 6500+ mass spectrometer (AB Sciex, Framingham, MA, USA). Two complementary chromatographic modes were employed to maximize metabolite coverage: hydrophilic interaction liquid chromatography (HILIC) and reversed-phase liquid chromatography (RPLC). For HILIC separation, a Waters UPLC BEH Amide column (2.1 mm × 100 mm, 1.7 µm) was maintained at 35 °C with an injection volume of 2 µL. Mobile phase A consisted of 90% water containing 2 mM ammonium formate and 10% acetonitrile, while mobile phase B was acetonitrile with 0.4% formic acid. The gradient elution was performed at a flow rate of 300 µL/min as follows: 85% B (0–1 min), 80% B (3–4 min), 70% B (6 min), 50% B (10–15.5 min), and 85% B (15.6–23 min). For RPLC separation, a Waters UPLC BEH C18 column (2.1 mm × 100 mm, 1.7 µm) was maintained at 40 °C with an injection volume of 2 µL. Mobile phase A was water containing 5 mM ammonium acetate, and mobile phase B was 99.5% acetonitrile. The gradient program at 400 µL/min was: 5% B (0 min), 60% B (5 min), 100% B (11–13 min), and 5% B (13.1–16 min). Throughout the analysis, samples were maintained at 4 °C in the autosampler.

Mass spectrometric detection was conducted using the 6500+ QTRAP system operating in positive/negative switching mode. For positive electrospray ionization (ESI+), the parameters were: source temperature 580 °C; ion source gas 1 (GS1) 45; ion source gas 2 (GS2) 60; curtain gas (CUR) 35; ion spray voltage (IS) +4500 V. For negative electrospray ionization (ESI−), the settings were: source temperature 580 °C; GS1 45; GS2 60; CUR 35; IS −4500 V. Data acquisition was performed using multiple reaction monitoring (MRM). Pooled quality control (QC) samples were injected periodically throughout the analytical sequence to monitor system stability and reproducibility.

For lipidomic profiling, analyses were conducted using a UHPLC system (LC-30AD, Shimadzu, Kyoto, Japan) coupled to a QTRAP 6500+ mass spectrometer (Sciex). Two separation modes were employed: reversed-phase chromatography and hydrophilic interaction chromatography. For C18 separation, a Phenomenex Kinetex C18 column (2.1 mm × 100 mm, 2.6 µm) was used at 45 °C. Mobile phase A consisted of 70% acetonitrile, 30% H_2_O, and 5 mM ammonium acetate; mobile phase B was isopropanol. The gradient program at 0.35 mL/min was: 20% B (0 min), 60% B (5 min), 100% B (13 min), and 20% B (13.1–17 min). For Amino separation, a Phenomenex Luna NH_2_ column (2.0 mm × 100 mm, 3 µm) was used at 40 °C. Mobile phase A contained 2 mM ammonium acetate in 50% methanol and 50% acetonitrile; mobile phase B consisted of 2 mM ammonium acetate in 50% acetonitrile and 50% water. The gradient at 400 µL/min was: 3% B (0–3 min), 3–100% B (3–13 min), 100% B (13–17 min), and 3% B (17.1–22 min). Samples were maintained at 10 °C during analysis. Mass spectrometry was performed using the 6500+ QTRAP system in positive/negative switching mode. ESI+ conditions were: source temperature 400 °C; GS1 50; GS2 55; CUR 35; IS +3000 V. ESI− conditions were: source temperature 400 °C; GS1 50; GS2 55; CUR 35; IS −2500 V. MRM was used for quantitative acquisition. Data processing was performed using MultiQuant (version 3.0.3, AB Sciex) for peak integration and Analyst software (version 1.7.1, AB Sciex) for data visualization. Metabolite identification was based on an in-house database provided by Applied Protein Technology, which contains retention times and MS/MS spectra for over 600 water-soluble metabolites and 1200 lipid species, confirmed using authentic standards where available. Peak areas were normalized to serum volume and internal standards to correct for instrument variability. QC samples were analyzed alongside biological samples to assess analytical variability. Metabolites and lipids with a coefficient of variation (CV) < 30% in QC samples were considered reproducibly measured and retained for subsequent statistical analysis. The MRM ion pairs for all detected metabolites and lipids are provided in the Supplementary Materials.

### 2.4. Statistical Analysis

Clinical and demographic characteristics of study participants were compared using one-way analysis of variance (ANOVA) and Student’s *t*-test for continuous variables, and the chi-square (χ^2^) test for categorical variables. Processed metabolomic and lipidomic data were analyzed using R software (version 4.3.0) with the ropls package (version 1.34.0) for multivariate statistical analysis. Specifically, Pareto-scaled principal component analysis (PCA) was initially performed to visualize overall sample distribution and detect potential outliers. Subsequently, orthogonal partial least-squares discriminant analysis (OPLS-DA) was employed to maximize group separation and identify discriminating metabolites and lipids. Model robustness and predictive performance were evaluated using 7-fold cross-validation and response permutation testing with 200 permutations. Variable importance in projection (VIP) values were calculated to quantify the contribution of each metabolite or lipid to the OPLS-DA classification model; variables with VIP > 1 were considered important contributors to group discrimination. For univariate analysis, Student’s *t*-test was applied to determine significant differences between two independent groups. Metabolites and lipids with *p*-values < 0.05, VIP > 1 (from OPLS-DA models) and fold change >1.5 or <0.67 were considered statistically significant. Pearson’s correlation analysis was performed to assess linear relationships between pairs of variables. To elucidate the biological relevance of the identified metabolites, pathway enrichment analysis was conducted using the Kyoto Encyclopedia of Genes and Genomes (KEGG) and the Human Metabolome Database (HMDB). Metabolic pathways with false discovery rate (FDR)-adjusted *p*-values < 0.05 were considered significantly enriched.

## 3. Results

### 3.1. Clinical Indicators Analysis

**Figure 1:**
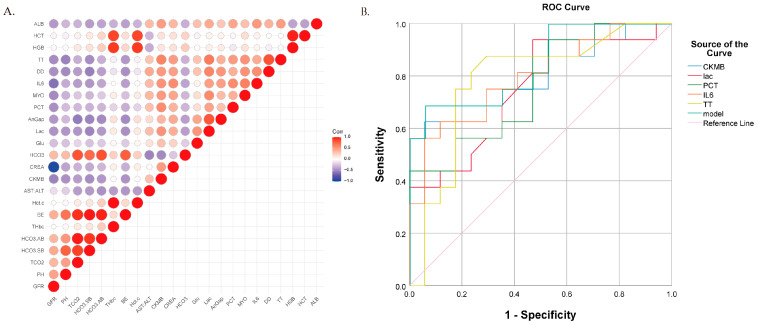
Clinical biomarker analysis in acute type A aortic dissection (ATAAD) and ATAAD with mesenteric malperfusion syndrome (ATAAD-MMPS). (**A**) Heatmap showing correlations between routine clinical biomarkers (*p* < 0.05) across patient groups. Red indicates positive correlation; blue indicates negative correlation; circle size reflects correlation strength. (**B**) Receiver operating characteristic (ROC) curves for the five most significant clinical biomarkers in ATAAD patients. Area under the curve (AUC) values: CK-MB (0.783), Lac (0.748), PCT (0.761), IL-6 (0.794), and TT (0.785). The vertical axis represents sensitivity, and the horizontal axis represents 1-specificity.

Clinical characteristics of the study cohorts are presented in Table 1. Patients with ATAAD-MMPS exhibited significant metabolic acidosis, hypercoagulability, and elevated inflammatory markers compared to uncomplicated ATAAD patients. As shown in Table 1, we compared routine clinical indicators between the ATAAD and ATAAD-MMPS groups. Multiple biomarkers exhibited significant differences (*p* < 0.05), including: (a) Marked alterations in acid–base parameters (HCO3.SB, BE; *p* = 0.002); (b) Elevated coagulation markers (TT, DD) in the MMP group; and (c) Inflammatory indicators (IL-6, PCT) with median values 3–5 times higher in the MMP group. After Pearson correlation analysis, subsequent multivariable logistic regression did not identify significant independent predictors of MMP. Receiver operating characteristic (ROC) analysis (Figure 1B) indicated moderate predictive performance of the selected biomarkers. Area under the curve (AUC) values ranged from 0.65 to 0.79, with interleukin-6 (IL-6) showing the strongest discriminative ability (AUC = 0.79; 95% CI: 0.64–0.94). Receiver operating characteristic (ROC) analysis for these clinical biomarkers showed moderate discriminative ability between ATAAD and ATAAD-MMPS (Figure 1B), with area under the curve (AUC) values ranging from 0.65 to 0.79, with interleukin-6 (IL-6) showing the strongest discriminative ability (AUC = 0.79; 95% CI: 0.64–0.94), although this remains within the moderate range and suggests limited utility as a standalone predictor.

### 3.2. Metabolomic Profiling Revealed Significant Differences Among ATAAD-MMPS, ATAAD, and Healthy Control Groups

**Figure 2:**
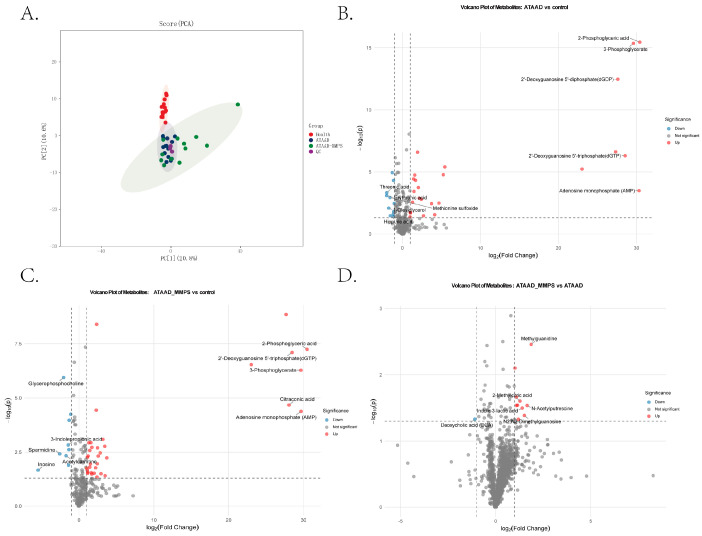
Metabolomic profiling distinguishes ATAAD and ATAAD-MMPS from healthy controls. (**A**) Principal component analysis (PCA) of metabolomic profiles from healthy controls (Ctr), uncomplicated acute type A aortic dissection (ATAAD), and ATAAD with mesenteric malperfusion syndrome (ATAAD-MMPS). (**B**–**D**) Volcano plot comparisons between ATAAD and Ctr (**B**), ATAAD-MMPS and Ctr (**C**), and ATAAD and ATAAD-MMPS (**D**). In each plot, individual metabolites are displayed with the horizontal axis representing log_2_(fold change, FC) and the vertical axis representing −log_10_(*p*-value). Dashed lines extending from the horizontal and vertical axes delineate the criteria for defining differentially abundant metabolites: *p* < 0.05 and FC ≠ 0 (i.e., log_2_FC ≠ 0). The fill color of circles corresponds to metabolite, with only the top five metabolites explicitly labeled. Circle outline colors indicate differential status: red represents increased levels in ATAAD (**B**) and ATAAD-MMPS (**C**,**D**), blue indicates decreased levels, and gray denotes no significant change. The top five significantly increased and decreased metabolites are listed for each comparison, selected based on absolute log_2_FC values in descending order.

To investigate the metabolic implications of mesenteric malperfusion and discover a new diagnostic biomarker, we performed comprehensive metabolomic profiling on serum samples from healthy controls (*n* = 13), patients with uncomplicated acute type A aortic dissection (ATAAD, *n* = 13), and patients with ATAAD complicated by mesenteric malperfusion syndrome (ATAAD-MMPS, *n* = 13). A total of 309 water-soluble metabolites were detected across the three cohorts. Principal component analysis (PCA) revealed a clear metabolic separation between the disease groups (ATAAD and ATAAD-MMPS) and healthy controls, indicating profound systemic metabolic alterations associated with aortic dissection (Figure 2A). Notably, the ATAAD-MMPS cohort exhibited greater inter-individual variability (score range: −5.61 to 38.42) and a less distinct separation from the uncomplicated ATAAD group, suggesting both shared and distinct metabolic features between these two patient populations. Volcano plot analysis identified 31 metabolites significantly altered in ATAAD patients compared to healthy controls, of which 22 were upregulated. These differentially abundant metabolites were primarily involved in energy metabolism, nucleotide metabolism, and amino acid metabolism. The five most significantly upregulated metabolites in ATAAD were 2-phosphoglyceric acid, 3-phosphoglycerate, 2′-deoxyguanosine 5′-diphosphate (dGDP), citraconic acid, and citicoline (Figure 2B). Compared to healthy controls, ATAAD-MMPS patients exhibited 57 upregulated and 10 downregulated metabolites. Strikingly, the magnitude of fold-change for energy metabolism intermediates was substantially greater in ATAAD-MMPS than in uncomplicated ATAAD. The magnitude of fold-change for several energy metabolism intermediates was substantially greater in ATAAD-MMPS than in uncomplicated ATAAD. For instance, 2-phosphoglyceric acid showed markedly higher levels in both disease groups compared to controls, with log_2_FC values of ~30.5 in ATAAD-MMPS and ~23.7 in uncomplicated ATAAD (Figure 2C). Such extreme log_2_FC values reflect the dynamic range of LC-MS/MS detection and may indicate metabolites that are present at very low or undetectable levels in control samples but become readily detectable in disease states. Direct comparison between ATAAD-MMPS and uncomplicated ATAAD revealed 11 significantly altered metabolites, 10 of which were upregulated in the MMPS group. Notably, this included a broad upregulation of tricarboxylic acid (TCA) cycle intermediates—citric acid, isocitric acid, cis-aconitic acid, trans-aconitic acid, and malic acid—suggesting enhanced TCA cycle flux or mitochondrial substrate accumulation under malperfusion conditions. Three metabolites were uniquely and markedly elevated in ATAAD-MMPS: gluconic acid, methylguanidine, and N-acetylputrescine (Figure 2D). In summary, these findings demonstrate distinct metabolic signatures associated with both ATAAD and ATAAD-MMPS in this pilot cohort. The metabolic disturbances characteristic of acute aortic dissection appears to be substantially exacerbated by the presence of mesenteric malperfusion. However, further studies focusing specifically on the gut ischemia–reperfusion continuum are warranted to fully elucidate the role of intestinal malperfusion in driving these metabolic alterations.

### 3.3. Mitochondrial Metabolism Was a Pivotal Determinant in AD Etiology

**Figure 3:**
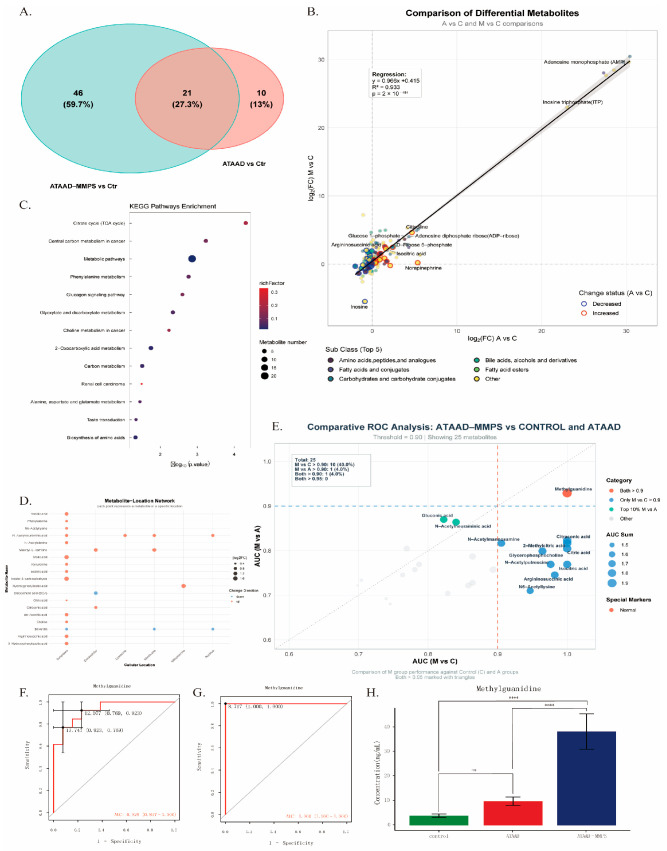
Metabolomic profiling reveals significant differences between ATAAD and ATAAD-MMPS patients compared to healthy controls. (**A**) Venn diagram illustrates differentially abundant metabolites in three comparisons: ATAAD vs. controls, ATAAD-MMPS vs. controls. (**B**) Linear regression plot comparing log2(fold change) of IDMs between ATAAD vs. controls (x-axis) and ATAAD-MMPS vs. controls (y-axis). Each point represents an IDM, with color indicating metabolite subclass (only top five subclasses are shown; others are categorized as “Other”). Circle outline color denotes differential status: red indicates increased levels in ATAAD vs. Ctr; blue indicates decreased levels; gray indicates no significant change. The top five upregulated and downregulated metabolites for each comparison are listed. The regression line equation, coefficient of determination (r^2^), and *p*-value are displayed. (**C**) KEGG pathway enrichment analysis of IDMs. Enrichment significance was determined using nominal *p* < 0.05 with Benjamini–Hochberg false discovery rate (FDR) correction for multiple testing. (**D**) Subcellular localization analysis of IDMs. Red indicates increased metabolites; blue indicates decreased metabolites. (**E**) Scatter plot of receiver operating characteristic (ROC) analysis for IDMs. Each point represents an IDM, with the horizontal axis showing AUC for ATAAD-MMPS vs. controls and the vertical axis showing AUC for ATAAD-MMPS vs. ATAAD. Dashed lines indicate the 0.9 threshold on both axes; points exceeding 0.9 in both comparisons are highlighted in red. (**F**,**G**) ROC curves for methylguanidine: F shows ATAAD-MMPS vs. ATAAD; G shows ATAAD-MMPS vs. controls. (**H**) Bar plot showing methylguanidine concentrations in controls, ATAAD, and ATAAD-MMPS groups. Data are presented as mean ± SEM. Normality was assessed by Shapiro–Wilk test; homogeneity of variance was evaluated by Bartlett’s test. Two-tailed unpaired Student’s *t*-test was used for comparisons with equal variances, **** *p* < 0.0001.

The tricarboxylic acid (TCA) cycle and amino acid metabolism are increasingly recognized as pivotal factors in the pathogenesis of aortic dissection. To dissect the metabolic alterations common to both ATAAD subtypes, we performed a comparative analysis of metabolites differentially abundant in each disease group relative to healthy controls. Venn diagram analysis identified 21 intersecting differentially abundant metabolites (IDMs) shared between ATAAD and ATAAD-MMPS (Figure 3A), suggesting a core metabolic signature associated with aortic dissection independent of malperfusion status. Regression analysis of log2-transformed fold changes revealed consistent dysregulation patterns across both AD subtypes. The affected metabolite subclasses encompassed amines; amino acids, peptides, and analogues; fatty acids and conjugates; carbohydrates and conjugates; bile acids, alcohols and derivatives; and fatty acid esters (Figure 3B). Notably, D-ribose 5-phosphate and isocitric acid displayed the most pronounced increases across both ATAAD subtypes, whereas inosine and methionine sulfoxide were among the most substantially decreased metabolites.

To elucidate the biological relevance of these shared metabolic alterations, we performed Kyoto Encyclopedia of Genes and Genomes (KEGG) pathway enrichment analysis on the 62 IDMs (38 metabolites with KEGG annotations; 13 enriched pathways). The TCA cycle emerged as the most significantly enriched pathway (Figure 3C). The top ten enriched pathways—including the TCA cycle; alanine, aspartate, and glutamate metabolism; glyoxylate and dicarboxylate metabolism; and butanoate metabolism—were predominantly associated with mitochondrial function. This conserved metabolic signature was observed consistently in both ATAAD subtypes, suggesting that mitochondrial dysfunction represents a central pathogenic mechanism in aortic dissection that is further exacerbated by the presence of malperfusion. Subcellular localization analysis of IDMs revealed widespread distribution of metabolic changes across cellular compartments, including cytoplasm, mitochondria, peroxisomes, and extracellular space (Figure 3D). The selective vulnerability of mitochondria suggests a predominant modulatory role in AD pathogenesis, as in comparison to the metabolite alterations observed in other organelles.

### 3.4. Methylguanidine Emerges as a Key Biomarker for ATAAD-MMPS

To identify potential diagnostic biomarkers distinguishing ATAAD-MMPS from uncomplicated ATAAD, we performed receiver operating characteristic (ROC) curve analysis on all intersecting differentially abundant metabolites (IDMs). Using a stringent AUC threshold of ≥0.9, only one metabolite—methylguanidine—demonstrated excellent discriminative capacity across both relevant comparisons (Figure 3E). Specifically, methylguanidine yielded an AUC of 0.923 for distinguishing ATAAD-MMPS from uncomplicated ATAAD, and an AUC of 1.000 for distinguishing ATAAD-MMPS from healthy controls (Figure 3F,G), indicating exceptional diagnostic performance. Quantitative metabolomic profiling revealed marked differences in methylguanidine concentrations across the three cohorts (Figure 3H). Methylguanidine levels were significantly elevated in both ATAAD-MMPS and uncomplicated ATAAD patients compared to healthy controls. Critically, a substantial and statistically significant increase was also observed when comparing ATAAD-MMPS directly with uncomplicated ATAAD, demonstrating specificity of this metabolite for the malperfusion complication. Collectively, these findings establish methylguanidine as a promising early diagnostic biomarker for ATAAD-MMPS, with the potential to facilitate timely identification of mesenteric malperfusion in patients presenting with acute type A aortic dissection.

### 3.5. Lipidomics Reveals Distinct Lipid Metabolism Profiles Among ATAAD-MMPS, ATAAD and Healthy Controls: Regardless of Mesenteric Malperfusion

**Figure 4:**
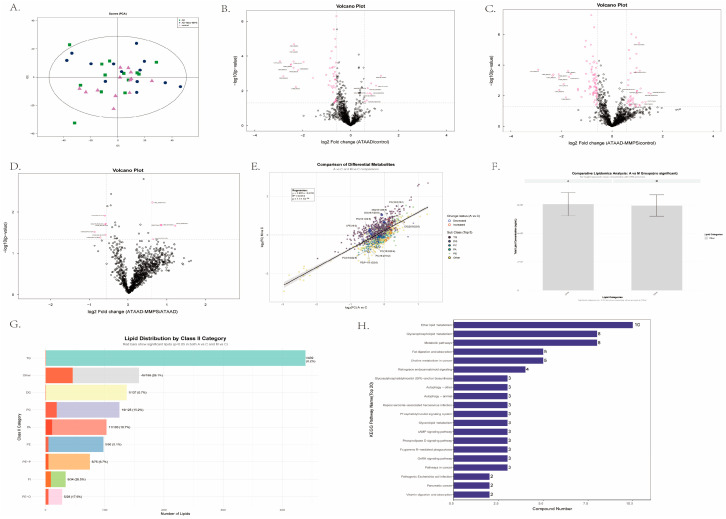
Lipidomic profiling reveals heterogeneity among ATAAD, ATAAD-MMPS, and healthy control groups. (**A**) Principal component analysis (PCA) of control (Ctr), ATAAD, and ATAAD-MMPS groups. (**B**–**D**) Volcano plot comparisons: ATAAD vs. Ctr (**B**), ATAAD-MMPS vs. Ctr (**C**), and ATAAD vs. ATAAD-MMPS (**D**). In each plot, individual lipids are displayed with log_2_ (fold change) on the x-axis and −log_10_(*p*-value) on the y-axis. Dashed lines extending from the horizontal and vertical axes delineate thresholds for defining differential lipids (*p* < 0.05 and FC ≠ 0). The top ten upregulated and downregulated lipids are listed for each comparison, ranked by absolute log_2_(FC) in descending order. For all volcano plots (**B**–**D**), log_2_FC values are calculated as (first named group)/(second named group) (e.g., ATAAD/Control), where positive values indicate higher levels in the first group and negative values indicate lower levels in the first group. (**E**) Linear regression plot comparing log_2_(FC) of intersecting differential lipids (IDLs) between ATAAD vs. Ctr (x-axis) and ATAAD-MMPS vs. Ctr (y-axis). Each point represents an IDL, with colors indicating lipid class according to the legend. Circle outline colors denote differential status: red represents upregulation, blue represents downregulation in ATAAD compared to controls. Gray indicates no significant change. The top five upregulated and downregulated lipid class are listed separately for each comparison, ranked by absolute log_2_(FC). Total IDLs are listed separately. The regression equation, r^2^, and *p*-value are displayed adjacent to the plot. (**F**) Comparison of overall lipid content between ATAAD and ATAAD-MMPS groups. Bar height represents lipid content, with colors indicating lipid classes according to the legend. Only classes with significant overall differences between groups are shown; others are categorized as “other”. Data are presented as mean ± SEM. (**G**) Comparison of relative lipid content distribution between groups by Class II category. Bar length represents relative lipid content, with colors indicating lipid classes. Only the top nine lipid categories with statistically significant differences in both ATAAD vs. controls and ATAAD-MMPS vs. controls (*p* < 0.05) are shown. Data are presented as mean ± SEM. Statistical tests were performed as described in panel (**F**). (**H**) KEGG pathway enrichment analysis of differential lipids. Bar plot displays significantly enriched pathways, with bar length representing the number of differential lipids enriched in each pathway. Only pathways with false discovery rate (FDR)-adjusted *p*-value < 0.05 are shown.

To further elucidate the underlying mechanisms, we conducted lipidomic profiling using the same sample set. Lipidomics analysis identified a total of 1197 lipid species. Principal component analysis (PCA) revealed overall similarity among the control, ATAAD-MMPS, and ATAAD groups (Figure 4A). More detailed analysis demonstrated differences at the individual lipid level across the three groups. In the comparison between ATAAD and controls, volcano-plot visualization highlighted 30 upregulated and 86 downregulated lipids in ATAAD (Figure 4B). The five most significantly altered lipid classes were triglycerides (TG), phosphatidylethanolamines (PE), lysophosphatidylcholines (LPC), phosphatidylcholines (PC), and fatty acids (FA). Notably, monoacylglycerol (MG) species—especially MG (22:5), MG (18:2), MG (20:4), MG (22:6), and MG (20:2)—showed the most pronounced downregulation in ATAAD. In contrast, lipids such as LPI(20:4), LPI(18:2), TG(56:1)-FA18:1, TG(56:1)-FA16:0, DG(20:0/20:2), PE(16:0/16:0), and PI(16:0/16:1) were significantly upregulated. When comparing ATAAD-MMPS to controls, 60 lipids were upregulated and 118 downregulated. MG (20:2) again exhibited marked downregulation (Figure 4C). Lipidomic comparison between ATAAD and ATAAD-MMPS revealed overall limited differences, with only 17 lipids showing significant reduction in ATAAD-MMPS (Figure 4D). Lipids such as PE(18:1/18:1), PE(O-18:0/20:3), PI(16:0/16:0), and TG(44:2)-FA16:1 were most prominently elevated, while ether-linked phosphatidylethanolamines (PE-P) were significantly downregulated—with PE(P-20:1/22:6), PE(P-20:1/20:4), PE(P-20:1/22:5), and PE(P-20:1/18:1) identified as the most reduced lipid species in ATAAD-MMPS.

## 4. Discussion

Acute type A aortic dissection (ATAAD) carries a mortality rate exceeding 40% within the first 24 h if left untreated, escalating to approximately 90% within three months [19]. The coexistence of mesenteric malperfusion syndrome (MMPS) further compounds this already grave prognosis [20]. For affected patients, timely surgical intervention remains the mainstay of treatment [21]; however, the underlying pathophysiological mechanisms of MMPS remain poorly understood, and effective early diagnostic biomarkers are lacking [22,23].

In this pilot study, we acknowledge that the sample size is relatively modest (*n* = 13 per group). The rarity of this condition is reflected in the epidemiological data: the estimated annual incidence of aortic dissection ranges from 2.8 to 4.3 per 100,000 population, among which Type A accounts for approximately 60% [1,24,25]. In patients with ATAAD, the prevalence of mesenteric malperfusion is approximately 4% [26], translating to an estimated incidence of less than 1 per 1,000,000 population. Consequently, prospective collection of well-characterized serum samples from this specific patient population poses substantial challenges. Given this inherent difficulty, our pilot study—despite its modest sample size—provides valuable preliminary evidence for understanding the pathogenesis of this devastating condition, and the findings should be considered hypothesis-generating.

To address this knowledge gap, we employed an integrated targeted metabolomics and lipidomics approach on identical clinical samples. This parallel strategy enabled direct correlation and reciprocal validation between the two omics datasets: metabolomics captured dynamic perturbations in energy metabolism, while lipidomics provided detailed characterization of lipid species alterations. Collectively, this multi-omics approach offers novel insights into the molecular underpinnings of ATAAD-MMPS and establishes a foundation for future biomarker discovery.

In this study, targeted metabolomic profiling revealed that mitochondrial metabolic perturbations represent a shared feature of both uncomplicated ATAAD and ATAAD-MMPS. Compared to healthy controls, both disease groups exhibited significant accumulation of tricarboxylic acid (TCA) cycle intermediates, including citric acid, isocitric acid, which as a strong signal indicating substantial metabolic network disturbances, often closely associated with reprogramming of energy metabolism. This pattern was further exacerbated in the ATAAD-MMPS group, with more pronounced elevations of these intermediates. These findings are consistent with prior studies demonstrating TCA cycle alterations in ischemic conditions, where mitochondrial electron transport chain impairment leads to upstream accumulation of cycle intermediates [27,28]. In the context of aortic dissection, mitochondrial dysfunction plays a critical role: impaired oxidative phosphorylation leads to insufficient energy supply for smooth muscle cells, compromising their ability to maintain a contractile phenotype. Furthermore, dysfunctional mitochondria release substantial amounts of reactive oxygen species (ROS), causing oxidative stress damage. ROS further damage mitochondrial DNA, creating a vicious cycle and activating downstream inflammatory pathways such as AP-1, thereby accelerating disease progression [29,30,31]. Another notable finding was the elevation of malonyl-CoA in the ATAAD-MMPS group. Malonyl-CoA inhibits carnitine palmitoyltransferase 1 (CPT1), the rate-limiting enzyme for mitochondrial fatty acid import, potentially impairing fatty acid oxidation and contributing to energy deficit in the setting of mesenteric malperfusion [32,33]. It is important to note, however, that serum metabolite levels alone cannot directly demonstrate mitochondrial dysfunction. These findings suggest an association between mitochondrial metabolic perturbations and ATAAD-MMPS, but further functional validation is needed.

Methylguanidine emerged as a particularly promising candidate biomarker in this pilot study. ROC analysis demonstrated strong discriminative capacity, with an AUC of 0.923 for distinguishing ATAAD-MMPS from uncomplicated ATAAD and an AUC of 1.000 for distinguishing ATAAD-MMPS from healthy controls. These results suggest that methylguanidine may serve as a valuable candidate for early identification of mesenteric malperfusion in ATAAD patients. However, it is important to acknowledge that these high AUC values may be influenced by the relatively small sample size in this pilot cohort; therefore, the diagnostic performance of methylguanidine requires confirmation in larger, independent prospective studies. The elevation of methylguanidine in ATAAD-MMPS may arise from multiple mechanisms. First, methylguanidine is known to be generated from creatinine via reactive oxygen species (ROS)-mediated oxidation in the liver [34,35]. Intestinal ischemia–reperfusion during mesenteric malperfusion likely generates substantial ROS, potentially enhancing methylguanidine production. Second, disruption of the intestinal barrier may allow bacterial products or even intact bacteria to enter the circulation; certain bacteria such as Pseudomonas stutzeri boliviensis possess enzymatic pathways for methylguanidine metabolism, raising the possibility of gut microbial contributions to circulating methylguanidine levels [36,37]. Notably, elevated methylguanidine has been reported in other pathological conditions, including chronic liver disease and uremia [38,39], and animal studies have demonstrated its neuroexcitatory properties [40,41]. These observations suggest that methylguanidine elevation may have broader pathophysiological implications beyond serving as a diagnostic marker.

In this study, metabolomic profiling identified a set of differentially abundant metabolites between ATAAD-MMPS and uncomplicated ATAAD. In contrast, lipidomic analysis revealed limited differences between these two groups. While both disease groups exhibited significant lipidomic changes compared to healthy controls, characterized by alterations in triglycerides (TG), phosphatidylethanolamines (PE), lysophosphatidylcholines (LPC), and phosphatidylcholines (PC), direct comparison between ATAAD-MMPS and uncomplicated ATAAD identified only 17 differentially abundant lipids. This finding suggests that lipidomic alterations are primarily associated with ATAAD itself rather than the presence of mesenteric malperfusion. Several factors may explain the divergent findings between metabolomics and lipidomics. First, at the biological level, metabolomics targets “hub metabolites” (e.g., TCA cycle intermediates) that are highly sensitive to perturbations, whereas lipids serve as structural membrane components with strong homeostatic regulation that resists dramatic changes [42]. Second, from a technical perspective, targeted metabolomics employs highly sensitive multiple reaction monitoring (MRM), while non-targeted lipidomics may suffer from ion suppression where high-abundance lipids mask subtle changes in low-abundance signaling lipids [43]. Third, temporal dynamics differ substantially: water-soluble metabolites turn over in seconds to minutes, whereas lipid turnover occurs over hours to days, making metabolomics more suitable for capturing acute pathological events. From a diagnostic perspective, these findings suggest that metabolomic profiling may be more suitable for detecting acute complications such as MMPS, whereas lipidomic analysis may offer complementary insights into the underlying disease process of ATAAD itself. Several limitations of this study should be acknowledged. First, the sample size was relatively small (*n* = 13 per group), which may limit the statistical power and generalizability of our findings. Post hoc power analysis was not performed given the exploratory nature of this pilot study. However, given the rarity and critical nature of ATAAD-MMPS—a condition with an estimated incidence of less than 5 per million population and extremely high early mortality—prospective collection of well-characterized serum samples from this specific patient population poses substantial challenges. Nonetheless, our findings should be considered exploratory and warrant validation in larger, multicenter cohorts. Second, the cross-sectional design precludes causal inferences. Longitudinal sampling, particularly before and after surgery, would better clarify whether metabolic changes—such as elevated methylguanidine—reflect reversible ischemia or ongoing organ damage. Third, although our multi-omics approach enabled comprehensive metabolic profiling, the absence of tissue-level validation limits our ability to link serum findings to specific organ pathologies. Future studies integrating paired tissue and serum samples may help address this gap. Fourth, while methylguanidine emerged as a promising diagnostic candidate, its mechanistic origin remains unclear—whether from ROS-driven creatinine oxidation, malperfusion-related renal impairment, or gut microbial metabolism under ischemia requires further investigation. Lastly, as a single-center study, our results may reflect population- or institution-specific factors. Multicenter validation across diverse populations is necessary before clinical translation.

Despite these limitations, the strength of this study lies in its focused investigation of an understudied, life-threatening complication of aortic dissection, leveraging integrated metabolomic and lipidomic profiling to generate novel hypotheses and identify candidate biomarkers with potential clinical utility.

## 5. Conclusions

In conclusion, this integrated metabolomics and lipidomics study reveals that ATAAD-MMPS is characterized by distinct metabolic perturbations involving mitochondrial dysfunction, which are superimposed on the broader lipidomic alterations associated with ATAAD itself. We identified methylguanidine as a novel and robust circulating biomarker that accurately distinguishes ATAAD-MMPS from uncomplicated ATAAD, holding promise for early diagnosis. These findings not only provide a basis for improved clinical management but also open new avenues for research into the pathophysiological role of mitochondrial metabolism and gut–kidney axis in this devastating condition.

## Figures and Tables

**Table 1:** Comparison of clinical biomarkers between ATAAD and ATAAD-MMPS groups (*p* < 0.05).

Parameter	ATAAD	ATAAD-MMPS	*p*-Value
ALB	40.237 ± 0.604	37.180 ± 1.160	0.041 *
GFR	90.052 ± 3.607	68.830 ± 5.453	0.011 *
PH	7.366 ± 0.010	7.319 ± 0.014	0.011 *
TCO2	22.863 ± 0.431	20.935 ± 0.682	0.024 *
HCO3.SB	24.337 ± 0.472	21.670 ± 0.654	0.002 **
HCO3.AB	25.716 ± 0.525	22.875 ± 0.788	0.005 **
THbc	147.95 ± 3.44	132.50 ± 4.62	0.011 *
BE	0.347 ± 0.616	−3.275 ± 0.908	0.002 **
Hct.c	45.342 ± 1.050	40.630 ± 1.412	0.012 *
AST: ALT	(0.860, 1.750)	(1.270, 2.285)	0.027 *
CK-MB	(8.800, 15.800)	(11.525, 44.400)	0.008 **
CREA	(69.200, 96.700)	(88.775, 122.125)	0.018 *
HCO3	(22.800, 25.100)	(16.875, 23.975)	0.015 *
Glu	(6.300, 8.800)	(7.550, 10.175)	0.037 *
Lac	(0.700, 2.000)	(1.450, 5.575)	0.006 **
AnGap	(8.500, 10.700)	(9.375, 15.375)	0.034 *
PCT	(0.027, 0.090)	(0.066, 0.481)	0.004 **
MYO	(27.73, 92.83)	(36.20, 376.50)	0.034 *
IL6	(26.73, 58.19)	(39.61, 220.35)	0.005 **
DD	(1.97, 11.47)	(4.48, 69.66)	0.004 **
TT	(16.200, 18.000)	(18.500, 22.600)	0.001 ***
HGB	144.00 ± 3.05	131.65 ± 4.14	0.021 *
HCT	43.610 ± 0.884	40.035 ± 1.145	0.018 *

Data are presented as mean ± standard deviation for normally distributed variables or median (Q1, Q3) for non-normally distributed variables. Statistical significance across groups was determined by one-way analysis of variance (ANOVA) with Bonferroni-adjusted post hoc comparisons for continuous variables, and chi-square test for categorical variables. Two-tailed Student’s *t*-test was used to assess significance between ATAAD and ATAAD-MMPS groups for numerical variables. Post hoc pairwise comparisons were performed for variables with significant overall *p*-values. Only parameters with *p* < 0.05 are shown. * *p* < 0.05, ** *p* < 0.01, *** *p* < 0.001.

## Data Availability

Data is provided within the manuscript or Supplementary Information Files.

## Supplementary Materials

The following supporting information can be downloaded at: https://www.mdpi.com/article/10.3390/metabo16040240/s1, Table S1: Clinical characteristics of study participants; Table S2: Complete list of detected metabolites (650 metabolites); Table S3: Complete list of detected lipids (2400 lipid species).

## Abbreviations

The following abbreviations are used in this manuscript:

Abbreviation	Full Term
ATAAD	Acute Type A Aortic Dissection
MMPS	Mesenteric Malperfusion Syndrome
ATAAD-MMPS	Acute Type A Aortic Dissection with Mesenteric Malperfusion Syndrome
TCA	Tricarboxylic Acid Cycle
ROC	Receiver Operating Characteristic
AUC	Area Under the Curve
LC-MS/MS	Liquid Chromatography–Tandem Mass Spectrometry
UHPLC	Ultra-High Performance Liquid Chromatography
PCA	Principal Component Analysis
OPLS-DA	Orthogonal Partial Least-Squares Discriminant Analysis
VIP	Variable Importance in Projection
FDR	False Discovery Rate
KEGG	Kyoto Encyclopedia of Genes and Genomes
HMDB	Human Metabolome Database
QC	Quality Control
CV	Coefficient of Variation
MRM	Multiple Reaction Monitoring
ROS	Reactive Oxygen Species
IL-6	Interleukin-6
PCT	Procalcitonin
LPS	Lipopolysaccharide
GFR	Glomerular filtration rate
